# Preferences Elicited and Respected for Seriously Ill Veterans through Enhanced Decision-Making (PERSIVED): a protocol for an implementation study in the Veterans Health Administration

**DOI:** 10.1186/s43058-022-00321-2

**Published:** 2022-07-20

**Authors:** Mary Ersek, Anne Sales, Shimrit Keddem, Roman Ayele, Leah M. Haverhals, Kate H. Magid, Jennifer Kononowech, Andrew Murray, Joan G. Carpenter, Mary Beth Foglia, Lucinda Potter, Jennifer McKenzie, Darlene Davis, Cari Levy

**Affiliations:** 1 Center for Health Equity and Promotion, Corporal Michael J. Crescenz VA Medical Center, 3900 Woodland Avenue, Annex Suite 203, Philadelphia, PA 19104 USA; 2 University of Pennsylvania Schools of Nursing and Medicine, Philadelphia, PA USA; 3 Sinclair School of Nursing and Department of Family and Community Medicine, University of Missouri, Columbia, MO USA; 4 Center for Clinical Management Research, VA Ann Arbor Healthcare System, Ann Arbor, MI USA; 5 Department of Family Medicine and Community Health, University of Pennsylvania Perelman School of Medicine, Philadelphia, USA; 6 Rocky Mountain Regional VA Medical Center, Aurora, CO USA; 7 University of Colorado Anschutz Medical Campus, Aurora, CO USA; 8 Corporal Michael J. Crescenz VA Medical Center, Philadelphia, PA USA; 9 University of Maryland School of Nursing, Baltimore, MD USA; 10 VA National Center for Ethics in Health Care, Washington, D.C., USA; 11 Department of Bioethics and Humanities, School of Medicine, University of Washington, Seattle, WA USA; 12 VA Purchased Long-Term Services and Supports, Geriatrics and Extended Care, D, Washington, .C USA; 13 Home-Based Primary Care Program, Office of Geriatrics and Extended Care, Washington, D.C., USA

**Keywords:** Life-sustaining treatment, Patient preferences, Patient decision-making, Long-term care, End-of-life care, Goals of care conversations, Implementation science, Audit with feedback, Facilitation

## Abstract

**Background:**

Empirical evidence supports the use of structured goals of care conversations and documentation of life-sustaining treatment (LST) preferences in durable, accessible, and actionable orders to improve the care for people living with serious illness. As the largest integrated healthcare system in the USA, the Veterans Health Administration (VA) provides an excellent environment to test implementation strategies that promote this evidence-based practice. The Preferences Elicited and Respected for Seriously Ill Veterans through Enhanced Decision-Making (PERSIVED) program seeks to improve care outcomes for seriously ill Veterans by supporting efforts to conduct goals of care conversations, systematically document LST preferences, and ensure timely and accurate communication about preferences across VA and non-VA settings.

**Methods:**

PERSIVED encompasses two separate but related implementation projects that support the same evidence-based practice. Project 1 will enroll 12 VA Home Based Primary Care (HBPC) programs and Project 2 will enroll six VA Community Nursing Home (CNH) programs. Both projects begin with a pre-implementation phase during which data from diverse stakeholders are gathered to identify barriers and facilitators to adoption of the LST evidence-based practice. This baseline assessment is used to tailor quality improvement activities using audit with feedback and implementation facilitation during the implementation phase. Site champions serve as the lynchpin between the PERSIVED project team and site personnel. PERSIVED teams support site champions through monthly coaching sessions. At the end of implementation, baseline site process maps are updated to reflect new steps and procedures to ensure timely conversations and documentation of treatment preferences. During the sustainability phase, intense engagement with champions ends, at which point champions work independently to maintain and improve processes and outcomes. Ongoing process evaluation, guided by the RE-AIM framework, is used to monitor Reach, Adoption, Implementation, and Maintenance outcomes. Effectiveness will be assessed using several endorsed clinical metrics for seriously ill populations.

**Discussion:**

The PERSIVED program aims to prevent potentially burdensome LSTs by consistently eliciting and documenting values, goals, and treatment preferences of seriously ill Veterans. Working with clinical operational partners, we will apply our findings to HBPC and CNH programs throughout the national VA healthcare system during a future scale-out period.

**Supplementary Information:**

The online version contains supplementary material available at 10.1186/s43058-022-00321-2.

Contributions to the literature

- This paper describes the application of implementation strategies in the Veterans Health Administration, the largest integrated healthcare system in the USA.
- The paper includes the planned methods to rigorously evaluate the processes and outcomes of two related quality improvement projects to enhance end-of-life decision-making for seriously ill patients.
- The quality improvement projects that are summarized in this paper will offer insights into effective ways to deploy and adapt audit with feedback and implementation facilitation to achieve clinical outcomes.

## Background

Patients living with serious illness are at risk for receiving potentially burdensome care that is not aligned with their values and goals [1–4]. Research shows that when people living with serious illness are provided information about life-sustaining treatments (LSTs), many express a desire to avoid LSTs, which do not improve quality of life and are burdensome rather than beneficial [5–7]. Receipt of potentially burdensome care at the end of life is more likely when clinicians fail to engage patients in open conversations about illness trajectories, the ineffectiveness of LSTs for people with advanced illness and debility, and the benefits of palliative-focused care [8]. These conversations are the first step in facilitating informed decision-making about LST preferences [9]. Translating these preferences into durable orders—that is, orders that remain in effect as the patient moves across healthcare settings and are not discontinued unless the patient or surrogate decision-maker changes them—is an effective way to honor patients’ choices about their care. Together, goals of care conversations and use of durable LST orders are associated with achieving preference-sensitive care [10–14], a higher likelihood of out-of-hospital death, and an increase in hospice enrollment [15–17].

A widely used approach for documenting LST preferences in durable medical orders that can be used within and across community healthcare settings is the Physicians Order for Life-Sustaining Treatment (POLST) paradigm. Originating in Oregon in the 1990s, the POLST paradigm is now a national movement, although each state oversees and directs its own implementation of the program. As a result, there are variations in the titles (e.g., Medical Orders for LST [MOLST]; Physicians Order for Scope of Treatment [POST]) as well as the laws and guidance governing their use and implementation [18]. Because of the variation across states, the term State Authorized Portable Orders (SAPOs) is often used to refer to these state-regulated LST processes and documents.

In 2017, the Veterans Health Administration (VA) National Center for Ethics in Health Care launched a national quality improvement program called the Life-Sustaining Treatment Decisions Initiative (LSTDI). The aim of the LSTDI is to promote personalized, proactive, patient-driven care for Veterans with serious illness by eliciting, documenting, and honoring their values, goals, and LST preferences. The initiative involves a national policy to standardize practices related to discussing and documenting goals of care and LST decisions, as well as tools, resources, education, and monitoring to support clinicians and facilities in making practice changes [19]. Under this VA program, goals of care conversations and LST preferences are documented in a standardized electronic health record (EHR) note and orders template (the LST template) that generates accessible and actionable medical orders. As durable orders, they do not automatically discontinue at discharge or when the patient crosses care settings within the VA. The VA LST template parallels a SAPO, which guides the process in community-based, non-federal healthcare entities.

The VA is the largest integrated healthcare system in the USA and cares for more than nine million Veterans at 170 VA medical centers [20]. Because of these characteristics, the VA is an ideal environment in which to explore, test, and tailor effective implementation strategies to promote consistent and sustainable evidence-based practices to improve care for patients living with serious illness and to honor their preferences for care at the end of life.

Because SAPOs and VA LST templates translate patient preferences for LSTs into medical orders that are immediately actionable, these approaches are appropriate for people with serious illness [18]. Two groups of Veterans who often live with serious illness and who are at high risk for receiving unwanted, potentially burdensome, costly care are those enrolled in Home Based Primary Care (HBPC) programs and those receiving care in community nursing homes (CNHs). Each year, VA HBPC programs care for more than 50,000 Veterans with complex, chronic illness who are too ill to visit outpatient clinics [21]. Annual mortality among HBPC enrollees is 39% [22]. Because of the high illness burden and limited life expectancy, goals of care conversations and Veterans’ LST preferences must be documented as soon as is feasible after admission to a VA HBPC program [23].

Since 1965, the VA has paid for eligible Veterans’ care in CNHs that are not part of the VA healthcare system. This care has been expanding dramatically to meet the needs of the aging Veteran population, and the average daily census in the VA CNH program is expected to grow by 80% by 2037 [24]. The VA CNH program is responsible for managing the quality of care and outcomes for nearly 41,000 Veterans annually [22]. In general, nursing home residents represent a population at high risk of hospitalization and death [25–27]. Despite the need to discuss and document LST preferences, many nursing home residents, including Veterans, are not actively involved or invited to engage in goals of care conversations and have their LST preferences documented in advance directives or translated into actionable medical orders [4, 28]. For community healthcare settings, these LST medical orders are SAPOs. VA CNH programs are well suited to facilitate SAPO completion because these teams, located at 170 VAMCs, oversee the care plans and monitor the quality of care every 45 days (more often, if needed) for all Veterans receiving VA-paid CNH care. The VA CNH field staff, typically comprised of nurses, social workers, program coordinators, and other licensed team members, are responsible for oversight of the Veteran’s care [29]. Because SAPOs are critical to ensuring that Veterans’ goals and treatment preferences are honored, efforts to promote completion of SAPOs and concordance with VA LST notes and orders templates should fully engage the VA CNH program leadership and staff.

To address challenges to conducting goals of care conversations and documenting Veterans’ LST preferences, an interdisciplinary team from three VA research centers developed the Preferences Elicited and Respected for Seriously Ill Veterans through Enhanced Decision-Making (PERSIVED) Quality Improvement (QI) program. Funded by the VA Quality Enhancement Research Initiative (QUERI), PERSIVED aims to improve decision-making and outcomes for Veterans with serious illness by increasing the number and quality of goals of care conversations and documentation of LST preferences in durable medical orders that can be honored in both VA (i.e., LST template) and non-VA settings (i.e., SAPOs). The purpose of this paper is to describe the goals and methods of the PERSIVED program, as well as early experiences in implementing two related but distinct projects.

## Design and methods

### Overview

PERSIVED is a partnership between VA-based implementation scientists and two operational offices: VA National Center for Ethics in Health Care and the Geriatrics and Extended Care Program. It involves rigorous process and outcomes evaluation using a stepped-wedge design applied to two related but separate projects. Project 1 focuses on Veterans receiving care through the VA’s HBPC program and project 2 involves Veterans enrolled in the VA CNH program.

PERSIVED builds upon earlier work conducted by the team under the auspices of the Long-Term Care (LTC) QUERI program, which examined early implementation of the LSTDI in HBPC programs and Community Living Centers (CLCs, VA-owned and operated nursing homes) [30]. The team used lessons learned from the LTC QUERI to inform PERSIVED. For example, LTC QUERI outcomes demonstrated that more intensive engagement with champions and implementation facilitation yielded better outcomes [31]. We also recognized that LST template completion rates for Veterans in VA HBPC varied greatly across programs [32] following the initial implementation of the LSTDI, which indicated significant opportunities for improvement. For this reason, we chose to continue our work with HBPC and to capitalize on our experiences and existing partnership with the national HBPC program office. In contrast to the wide range of LST template completion in HBPC programs at the end of the LTC QUERI, mean LST template completion rates across all VA CLCs exceeded 80% [31]. This high baseline completion rate limited the potential impact of additional implementation efforts, and thus, we turned our attention to the VA CNH program, where LST template completion ranges from 2 to 87% (Internal VA data, 2022).

### Guiding frameworks

Two frameworks guided the development of PERSIVED and its evaluation: RE-AIM [33, 34] and the Tailored Implementation in Chronic Disease (TICD) framework [35]. RE-AIM examines five domains—*R*each, *E*ffectiveness, *A*doption, *I*mplementation, and *M*aintenance—and is used to guide evaluation of processes and outcomes. RE-AIM can also be used to identify and measure factors that facilitate or hinder success in achieving broad uptake and success of the intervention. The TICD is a wide-ranging framework that guides understanding of determinants of successful implementation of evidence-based programs in healthcare settings. The TICD consists of seven factors: Guidelines; Health Professionals; Patients; Professional Interactions; Incentives and Resources; Capacity for Organizational Change; and Social, Political, and Legal factors.

### Description of the individual projects

Table 1 summarizes key components of the two projects. The Project 1 team works directly with HBPC clinicians (e.g., social workers, nurses, and providers) to increase the number and quality of goals of care conversations and with prescribing practitioners (i.e., physicians, advance practice nurses, and physician assistants) to increase the number of HBPC patients with completed VA LST notes and accompanying orders.

**Table 1:** Summary of projects

Characteristic	Project 1: Home Based Primary Care programs	Project 2: Community Nursing Home Programs
**Aim**	Equip clinicians with data, tools, and processes to consistently document LST preferences among seriously ill Veterans and convert their preferences into actionable orders to promote goal-concordant care	Equip clinicians with data, tools, and processes to consistently document LST preferences among seriously ill Veterans, convert Veterans’ preferences into actionable orders that cross VA and non-VA settings to promote goal-concordant care, and communicate preferences among VA and non-VA clinicians
**Total number of sites enrolled over the project**	12	6
**Number of sites per cohort (3 cohorts)**	4	2
**Criteria for site selection**	1. Programs with low baseline LST template completion (≤ 50% of patients) 2. Geographic diversity	1. Geographic diversity 2. Program diversity: • Program size • Number of contracted community nursing homes • Rural/urban settings

Eligible HBPC programs include all those with low LST completion rates (≤ 50% of patients). Geographic diversity is a secondary inclusion criterion. The PERSIVED team assembles a list of eligible programs and shares it with the national HBPC program manager, who personally reaches out to selected programs to offer the opportunity to participate. Recruitment ceases when 12 programs have accepted the invitation.

Project 2 is being conducted in six VA CNH programs and focuses on Veterans receiving long-term care (as opposed to short-stay or post-acute care). The approaches for Project 2 differ from Project 1 for a few reasons. First, VA CNH teams oversee and coordinate the care that Veterans in CNHs receive but they do not provide direct care. Second, most VA CNH teams are comprised of registered nurses and social workers but do not include practitioners who are authorized by VA policy to establish LST plans and write LST orders. As a result, VA CNH team members usually are not involved in goals of care conversations or in completing LST templates or SAPOs. For this reason, QI efforts for Project 2 focus on (1) partnering with VA practitioners (e.g., in primary care, specialty clinics, and palliative care teams) to conduct goals of care conversations and complete VA LST notes and orders, as well as SAPOs when appropriate; (2) monitoring, documenting, and communicating the existence of LST orders across all settings where the Veteran receives care; and (3) recognizing and communicating changes in Veterans’ preferences and/or discrepancies among LST notes and orders across settings.

Because the PERSIVED team has not previously worked with the VA CNH program, we limited Project 2 to six sites compared with the 12 sites in Project 1. Given the small number of sites, we focused recruitment on CNH program size (large vs. small) and geographically diverse locations rather than LST completion rates, although we avoided sites with high rates of LST template completion (i.e., > 60%).

Participating sites for both projects are recruited and enrolled in three cohorts using a stepped wedge design.

### Intervention

**Fig. 1:**
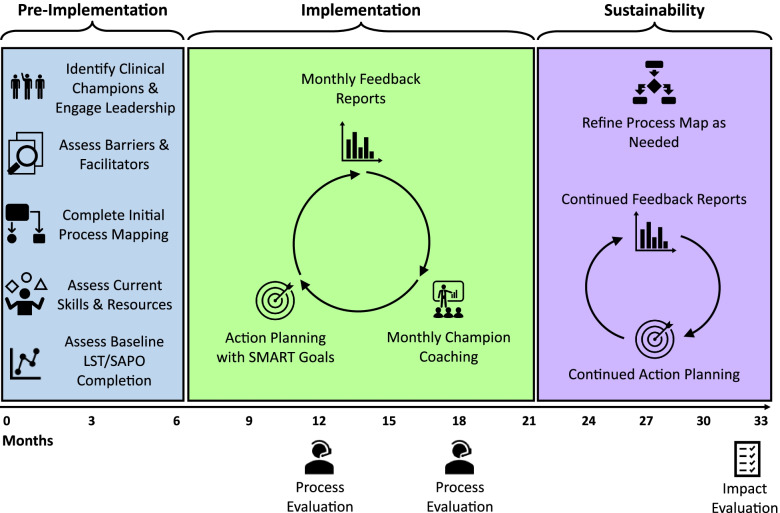
Implementation plan and timeline

The basic intervention for both projects is similar and consists of three phases: (1) 5–6-month *pre-implementation* phase, (2) 15–16-month *implementation* phase, and (3) 12-month *sustainability* (maintenance) phase (Fig. 1). Taken together, all three phases comprise the intervention, which is initiated anew with each of the three cohorts. Following the sustainability phase, we will use the findings generated during our process and outcomes evaluation to disseminate the intervention to HBPC and CNH programs throughout the VA healthcare system during a *scale-out* period.

#### Pre-implementation phase

The pre-implementation phase focuses on activities to prepare site personnel to engage in QI activities. It begins with a virtual kick-off meeting with site leadership and HBPC/CNH team members. At this meeting, PERSIVED team members explain the project, delineate roles, outline expectations of both the PERSIVED team and the site team, and answer questions about the project. Following the kick-off meeting, we conduct additional virtual or, if possible, in-person meetings to (1) engage site leadership and teams and identify key local team members, including site champions; (2) assess barriers and facilitators to project adoption; (3) develop process maps of existing workflows; and (4) measure baseline LST template/SAPO completion rates. Based on our findings, we develop a flexible, tailored implementation facilitation plan for each site, working closely with champions and other team members (Additional file 1: Examples of Barriers and Strategies to Address Barriers to PERSIVED Implementation).

Using criteria and methods developed in the LTC QUERI, we work with local leadership to identify and recruit the site champions [30, 36] (Additional file 2: PERSIVED Site Champion Description and Assessment Tool). Preparation of champions includes assessment of their confidence and skills around key practice elements (e.g., conducting goals of care conversations; understanding and completion of LST/SAPO notes and orders; understanding of audit with feedback and QI activities). Based on the findings of the assessment, PERSIVED coaching teams provide training and support to increase champions’ skills and confidence, which continues into the implementation phase as needed.

Another key pre-implementation activity is to conduct a contextual inquiry using qualitative, semi-structured interviews with key stakeholders (leaders, site champions, and clinicians). Using the TICD framework, we apply rapid qualitative analysis methods [37–39] to identify barriers and facilitators to conducting goals of care conversations, documenting LST preferences, and communicating these preferences across VA and non-VA settings as appropriate.

Also included in pre-implementation is process mapping, which is an ideal method for mapping complex healthcare workflows. Maps can be used as a communication tool for engaging with healthcare providers as part of the QI process [40, 41]. Process mapping interviews are conducted virtually, beginning with a pre-existing “sketch” of the process obtained from clinical operations partners or constructed from pre-implementation interviews conducted with key stakeholders. These steps include all assessment and decision-making processes involved in the admission, follow-up, and oversight activities for Veterans in the HBPC and VA CNH programs. Also mapped are existing activities related to conducting goals of care conversations, completing LST templates, and checking for existing LST documentation and SAPO completion. Following the initial sessions, team members use Microsoft Visio software to generate a draft process map and obtain feedback from sites to correct and refine the map. Process maps are used iteratively during implementation facilitation to track progress and modify the implementation plan as needed (Additional file 3: Example of Process Map).

#### Implementation phase

The implementation phase runs from months 6 to 21. Its activities are anchored by two key implementation strategies: audit with feedback and implementation facilitation. Audit with feedback has been tested through many randomized controlled trials and shown to have a generally positive, though modest, absolute effect of increasing the likelihood of achieving desired behavior change [42, 43]. Audit with feedback also has been used to augment education to sustain high rates of completion of actionable medical orders in seriously ill populations [44]. Brehaut et al. [45] reviewed the literature on audit with feedback and made 15 recommendations for optimizing its effectiveness. The PERSIVED team incorporated several of these recommendations including presenting data that drives actions which are under the clinicians’ control; keeping visual information simple and closely linking the display with the summary message; and providing regular, ongoing feedback rather than “one-off” feedback.

The second strategy is implementation facilitation, which Kirchner et al. describe as “a multifaceted strategy that applies a variety of discrete strategies…depending on what is needed given the context and characteristics of those that are providing (clinicians) and receiving (patients) the innovation” [46, 47]. Facilitation strategies incorporate a broad array of implementation approaches, including recruiting and coaching site champions and using Plan-Do-Study-Act (PDSA) cycles. Many of these have been tested in multiple trials and quasi-experimental studies, with evidence of effectiveness [47], as facilitation allows for iterative, tailored approaches to implementation [46, 47]. Incorporating implementation facilitation strategies also aligns our methods with advice from implementation scientists who recommend that audit with feedback be coupled with additional approaches, specifically to support action planning [42, 47].

Using these two strategies, the implementation phase involves monthly coaching sessions with the champions aimed at increasing their self-efficacy and effectiveness in guiding local teams and partners to meet the project goals. Facilitation begins with the creation of an implementation planning guide specific to the PERSIVED intervention, which is used to guide initial implementation planning meetings. Regular meetings with site champions involve monitoring the implementation plan, discussing and documenting progress, identifying barriers, helping problem-solve and identify solutions, modifying the plan as needed, and providing support, encouragement, and positive reinforcement [41].

PERSIVED team members also review monthly feedback reports with champions. The reports use formats similar to those developed in the LTC QUERI [48] and include rates of LST template completion at each site (Additional file 4: Example of Feedback Report). Based on success in a previous LTC QUERI project [36], we also include current site rosters that allow the champions and other clinicians to readily identify Veterans without documentation and prioritize these Veterans for goals of care conversations. Monthly coaching meetings include support and guidance for champions in developing action plans and monitoring progress using SMART (Specific-Measurable-Achievable-Relevant-Time Bound) goals [42, 47].

#### Sustainability/maintenance phase

During the sustainability phase (months 21–33), participating HBPC and CNH sites will continue to receive the feedback reports. In contrast to the implementation phase, site champions will receive limited coaching and instead be expected to maintain the project independently, including refining process maps as needed and engaging in action planning (Fig. 1).

#### Scale-out phase

The eventual goal is to achieve a broad-based scale out of the projects to all CNH and HBPC programs, with the potential to improve care for over 40,000 Veterans annually in VA CNH programs and over 50,000 Veterans who receive care in HBPC annually. In this final, post-intervention step, we will analyze process and outcomes data to identify the most effective strategies for successful implementation of our evidence-base practices. Based on these findings, we will develop interactive, web-based toolkits for both projects that are modeled after the successful VA Patient Engagement Toolkit (https://go.usa.gov/xUtdV) [49]. The toolkit will include a virtual playbook outlining the steps necessary to implement the practices in other HBPC and CNH programs. The playbook will begin with process maps developed during implementation augmented with an interactive timeline. This will allow adopters to begin their journey based on where they currently are in the implementation process. Links within the timeline will provide 3- to 5-min coaching podcasts based on key steps identified during implementation coaching sessions. High-performing sites will share strategies for overcoming barriers to implementation at each implementation milestone.

### Evaluation plan

We are using mixed quantitative and qualitative data analyses to examine our implementation success across components of the RE-AIM framework. The quantitative measures are listed in Table 2 and described below.

**Table 2:** RE-AIM process and outcome measures

RE-AIM domain	Measure	Unit of analysis
Reach	• Proportions of nonwhite Veterans with LST templates are similar to the proportion of white, non-Hispanic Veterans with LST templates	• HBPC program
	• Proportions of nonwhite Veterans with SAPOs are similar to the proportion of white, non-Hispanic Veterans with SAPOs	• CNH program
Effectiveness	Advance Care Plan (VA LST template or SAPO)	
	• % Veterans with LST template completed	• HBPC program
	• % Veterans with completed SAPO	• CNH program
	Concordance between documented preferences and care received by Veteran at end-of-life	
	• % Decedents w/comfort goal with hospice in the last 90 days of life	• Veteran
	• % Decedents w/comfort goal without ICU admission in the last 90 days of life	• Veteran
Implementation	• Proportion of HBPC programs that achieve >80% LST completion rates by the end of the implementation phase	• HBPC program
	• Proportion of VA CNH programs that achieve >80% of SAPO/LST medical order rates by the end of the implementation phase	• CNH program
Maintenance	• Proportion of HBPC programs that maintain ≥ 80% of patients with LST documentation at the end of 1-year sustainability phase	• HBPC program
	• Proportion of VA CNH programs that maintain ≥ 80% of patients with SAPO/medical order documentation at the end of 1-year sustainability phase	• CNH program

#### Reach

In the RE-AIM framework, Reach refers to the success of the intervention in reaching the targeted population [50]. Our target is that all Veterans in the participating CNH and HBPC programs are given an opportunity to discuss their goals and document their LST preferences. Several studies have documented that nonwhite patients, including Veterans, are less likely to engage in goals of care discussions and have lower rates of LST and SAPO completion [51–53]. Eliminating racial and ethnic disparities in the LST template and SAPO completion is important; thus, to evaluate the Reach of the PERSIVED interventions, we will compare the proportions of nonwhite/Hispanic Veterans with LST templates (HBPC) or SAPOs (CNH) vis-à-vis the white, non-Hispanic Veterans. Our goal is that there are no statistically significant differences in the LST template and SAPO completion between the groups.

#### Effectiveness

Program effectiveness will be assessed using two metrics. The primary metric is evidence of an Advance Care Plan, which is a National Quality Forum-endorsed measure and a CMS Meaningful Measure [54]. Although the CMS measure includes any documentation of an advanced care plan in the medical record, we specifically operationalize this metric as the proportion of Veterans enrolled in the HBPC program with a completed LST template in the VA EHR or proportion of Veterans enrolled in the CNH program with a completed SAPO in the CNH medical record.

The analytic plan to evaluate success in increasing LST plans and orders at sites is similar to the one we used in the LTC QUERI [31]. Because many sites are not chosen or assigned to a cohort at random, we will need to control for selection bias in our outcomes analysis. To achieve this balance, we will generate a predicted value or score, following the approach of Byrne et al. [55, 56] to match participating VA CNH and HBPC programs with comparison programs (i.e., HBPC and CNH programs that did not participate in either project). We will match intervention to comparison sites using Euclidean distance calculated between scores for each site derived by estimating principal components analysis (PCA), a factor analytic approach that uses a large number of variables to generate a predicted value or score.

After matching, we will use interrupted time series/segmented regression analysis (ITS/SRA) [57] to evaluate effectiveness using Advance Care Plan (i.e., LST template or SAPO) as the dependent variable. We will conduct separate analyses for Project 1 and Project 2. Outcomes for intervention sites will be compared to baseline data during the baseline pre-implementation period. The initiation of audit with feedback and facilitation represents the interruption in each analysis. ITS/SRA has been used frequently for quasi-experimental study designs, including previous analyses by the LTC QUERI [31].

The second metric is the concordance between documented preferences and care received at end of life, which reflects the ultimate goal of our evidence-based practices — to achieve patient-centered/patient-directed care by honoring Veterans’ values and care preferences in the treatments that we provide [58]. We operationalize this measure by examining the care provided to Veterans who have a goal of “to be comfortable” documented on either a VA LST template or “comfort measures only” on a SAPO or facility medical order. Specifically, we will examine the associations between a documented goal of comfort and (1) receipt of hospice care (yes/no) and (2) ICU admissions in the last 90 days of life (yes/no). These outcomes are aligned with quality indicators and are associated with higher family ratings of end-of-life care [1, 59]. We hypothesize that a documented comfort goal will be significantly associated with higher hospice use and fewer ICU admissions. We chose ICU admissions instead of hospitalizations because an earlier study found that most hospitalizations among frail nursing home residents with comfort orders were unavoidable and/or occurred to keep the resident comfortable [60]. The analysis plan to evaluate care concordance is similar to the one used in the LTC QUERI. We will model the two binary outcomes using multivariate logistic regression.

#### Implementation

Our implementation goal is to increase the consistent documentation of LST preferences for veterans served by the participating programs. We set a benchmark of 80% or greater LST template or SAPO completion rates. We will deem the PERSIVED program as successful if all participating HBPC and CNH programs meet this criterion at the end of their respective implementation phases (Fig. 1).

#### Maintenance

We will evaluate the sustainability of the intervention using a similar approach as described above. We will define success in maintenance/sustainability as the percentage of participating HBPC and CNH programs that are able to maintain ≥ 80% LST template or SAPO completion at the end of the sustainability phase. Our goal is that 100% of participating HBPC and CNH programs sustain these high completion rates. Participating programs that do not achieve the benchmark by the end of the implementation phase will be given a 2-month grace period at the beginning of the sustainability phase to meet the benchmark; programs that do not meet the benchmark by that time will be counted as failures in both implementation and sustainability regardless of their completion rates at the end of the sustainability phase.

#### Process evaluation

In addition to assessing RE-AIM outcomes, we will also conduct process evaluations during the implementation phase, at months 12 and 18. The evaluation team will conduct interviews with site champions to (1) guide adaptation of champion coaching activities, (2) address new and ongoing barriers, (3) review and revise process maps, (4) prepare sites for sustainability following the period of active implementation, and (5) develop the interactive toolkit and playbooks for use during scale-out. The process evaluation will help us assess and understand the fidelity of implementation, specifically, the degree to which champions share the feedback reports with others, engage in action planning, and meet targets using SMART goals (Adoption and Implementation). We will also evaluate champions’ self-efficacy and perceived effectiveness in participating in the intervention (Effectiveness) (Additional file 2: PERSIVED Site Champion Description and Assessment Tool).

#### Data sources and collection

Sources for our quantitative data will be derived from the VA’s Corporate Data Warehouse, a centralized repository that houses VA clinical, administrative, and financial data systems. To measure non-VA hospice use and ICU admissions (Table 2: Effectiveness), we will examine VA inpatient claims, Medicare fee-for-service inpatient claims, and the VA Program Integrity Tool. For ICU admissions, we will use bed section codes for VA hospitalizations and revenue center codes for Medicare-related hospitalizations. By including Medicare claims data into the database, we can more accurately measure healthcare utilization for Veterans who receive care outside of the VA.

Additional qualitative and quantitative data will be collected from evaluation teams from all three VA research centers that are involved in PERSIVED. They will operate independently of the facilitation team and conduct evaluation through in-person and virtual interviews. Some data will be collected during monthly coaching calls and uploaded to preset templates using the VA’s REDCap® secure web-based application. As described earlier, semi-structured interview guides are used for baseline interviews and process evaluations with HBPC and CNH staff and champions to capture barriers and facilitators to adoption of the evidence-based practice. These interviews are captured on Teams video-recording function and rough transcripts are generated automatically. The analytic team then uses rapid qualitative analysis methods to summarize results within and across sites [37–39].

## Discussion

### Early experiences: identifying strengths and challenges

We encountered some anticipated and unanticipated challenges early in the project, most prominently those involving the COVID-19 pandemic and recruitment of site champions. We also found that we could draw on our previous work and established relationships during the LTC QUERI to overcome several of these challenges.

Not surprisingly, some of the biggest obstacles we faced were the result of the COVID-19 pandemic, which overwhelmed the healthcare system and worsened existing challenges. For example, in-person oversight visits by VA CNH teams were halted and many HBPC visits transitioned to telehealth platforms [61]. Clinicians in both projects identified that conducting goals of care conversations by phone or online via videoconference was difficult given the sensitive nature of the topic. Nonetheless, they also recognized that the pandemic increased the urgency to have these conversations with patients and families. Leadership may have varying priorities and the LSTDI might not be a priority for their facility at this time. The pandemic may also have contributed to some reluctance on the part of sites to participate. For example, one of the CNH programs and two HBPC programs we approached declined participation, citing staff shortages and competing priorities. While this will serve as a limitation in our analysis, we were able to leverage our strong established relationships with the national operations leadership of both programs to identify other appropriate sites to enroll.

A second challenge involves the recruitment of champions at some of the sites. In addition to the stresses of the pandemic, the long-term engagement with PERSIVED (32–33 months) can seem daunting. To address this hurdle, we mapped out the champions’ expected activities along with time estimates and showed potential champions that their commitment would consist of approximately 1 to 2 h per month. Recruiting more than one champion at each site further minimized the time commitment of individual champions.

Other threats to the sustainability of the PERSIVED program are the ever-changing priorities of the VA healthcare system as well as local and national VA initiatives. For example, the rollout of Cerner—VA’s new electronic medical record system—will result in changes in how goals of care conversations, advance care planning, and LST preferences are documented and communicated within the system. Both projects plan to engage at least one site where the new electronic health record will be implemented early in the national rollout.

### Summary

The PERSIVED program supports the implementation and dissemination of a complex intervention to elicit, document, and honor seriously ill Veterans’ goals, values, and LST preferences for end-of-life care. Our evidence-based practice—structured goals of care conversations and documentation of LST preferences in durable, actionable medical orders—as well as our measures of effectiveness, are widely recognized as indicative of quality healthcare. As such, PERSIVED can contribute to patient care by testing the effectiveness of our intervention. The program can also support implementation science by reporting results of audit with feedback coupled with facilitation and exploring the reach, adoption, implementation, and sustainability of these approaches.

## Supplementary Information

**Additional file 1.** Examples of Barriers and Strategies to Address Barriers to PERSIVED Implementation. Description: Planning document anticipating potential barriers and implementation strategies to address such barriers.

**Additional file 2.** PERSIVED Site Champion Description and Assessment Tool. Description: A description of responsibilities expected of clinical champions & the assessment tool to measure respondent’s confidence in areas that are important to a successful champion.

**Additional file 3.** Example of Process Map. Description: A process map (redacted) developed with a participating VA CNH program during the pre-implementation phase.

**Additional file 4.** Example of Feedback Report. Description: An example of the feedback reports that are generated each month for participating sites.

## Data Availability

Because this work is being done as quality improvement, data will only be available from the authors on request and after approval by the authorizing officials.

## Abbreviations

ACP: Advance care planning
CNH: Community Nursing Home
HBPC: Home Based Primary Care
EOL: End of life
ITS/SRA: Interrupted time series/segmented regression analysis
LST: Life-sustaining treatments
LSTDI: VA Life-Sustaining Treatment Decisions Initiative
LTC: Long-term care
MOLST: Medical Orders for Life-Sustaining Treatments
OLS: Ordinary least squares
PDSA: Plan-Do-Study-Act
PERSIVED: Preferences Elicited and Respected for Seriously Ill Veterans through Enhanced Decision-Making
POLST: Physicians’ Order for Life-Sustaining Treatments
QI: Quality Improvement
QUERI: Quality Enhancement Research Initiative
RE-AIM: *R*each-*E*ffectiveness-*A*doption-*I*mplementation-*M*aintenance
SAPO: State Authorized Portable Orders
SMART: Specific-Measurable-Achievable-Relevant-Time Bound
TICD: Tailored Implementation in Chronic Disease
VA: Veterans’ Health Administration
