# Correction: Preferences Elicited and Respected for Seriously Ill Veterans through Enhanced Decision-Making (PERSIVED): a protocol for an implementation study in the Veterans Health Administration

**DOI:** 10.1186/s43058-023-00416-4

**Published:** 2023-03-14

**Authors:** Mary Ersek, Anne Sales, Shimrit Keddem, Roman Ayele, Leah M. Haverhals, Kate H. Magid, Jennifer Kononowech, Andrew Murray, Joan G. Carpenter, Mary Beth Foglia, Lucinda Potter, Jennifer McKenzie, Darlene Davis, Cari Levy

**Affiliations:** 1 Center for Health Equity and Promotion, Corporal Michael J. Crescenz VA Medical Center, 3900 Woodland Avenue, Annex Suite 203, Philadelphia, PA 19104 USA; 2 University of Pennsylvania Schools of Nursing and Medicine, Philadelphia, PA USA; 3 Sinclair School of Nursing and Department of Family and Community Medicine, University of Missouri, Columbia, MO USA; 4 Center for Clinical Management Research, VA Ann Arbor Healthcare System, Ann Arbor, MI USA; 5 Department of Family Medicine and Community Health, University of Pennsylvania Perelman School of Medicine, Philadelphia, USA; 6 Rocky Mountain Regional VA Medical Center, Aurora, CO USA; 7 University of Colorado Anschutz Medical Campus, Aurora, CO USA; 8 Corporal Michael J. Crescenz VA Medical Center, Philadelphia, PA USA; 9 University of Maryland School of Nursing, Baltimore, MD USA; 10 VA National Center for Ethics in Health Care, Washington, D.C USA; 11 Department of Bioethics and Humanities, School of Medicine, University of Washington, Seattle, WA USA; 12 VA Purchased Long-Term Services and Supports, Geriatrics and Extended Care, Washington, D.C USA; 13 Home-Based Primary Care Program, Office of Geriatrics and Extended Care, Washington, D.C USA

**Correction: Implement Sci Commun 3, 78 (2022)**

**https://doi.org/10.1186/s43058-022-00321-2**

Following the publication of the original article [1] the authors requested to update the “Competing interests” section as follows:

“The authors declare that Anne Sales is co-Editor-in-Chief of the journal."
