# Supplementary material for: Preferences Elicited and Respected for Seriously Ill Veterans through Enhanced Decision-Making (PERSIVED): a protocol for an implementation study in the Veterans Health Administration
Source: Implement Sci Commun. 2022 Jul 20;3:78. doi: 10.1186/s43058-022-00321-2 (PMC9296899; doi:10.1186/s43058-022-00321-2)
Supplement: Supplementary file 1 — Additional file 1. Examples of Barriers and Strategies to Address Barriers to PERSIVED Implementation. Description: Planning document anticipating potential barriers and implementation strategies to address such barriers. [file 43058_2022_321_MOESM1_ESM.pdf]

Examples of Barriers and Strategies to Address Barriers to PERSIVED Implementation[1]		
**Category**	**Examples of specific barriers that may be encountered**	**Examples of implementation strategies to address barriers**
Clinical Champion (CC)	CC is uncertain or misguided about their role	• Use adapted LTC QUERI "Site Champion Description"[2] to recruit CC
	CC lacks confidence and/or skills to be effective	• Use CC Assessment of Effectiveness Tool and coaching to enhance CC effectiveness
	CC is re-assigned other duties or leaves the facility/VA	• Recruit and coach >1 CC where possible
VA CNH and HBPC Team Members	Team members lack the confidence and/or skills to conduct GoCC and/or complete a SAPO[3,4]	• Offer team members NCEHC-developed GoCC training by PERSIVED clinical experts<br>• Offer role modelling and debriefing opportunities with PERSIVED clinical experts
	Team members lack time to conduct GoCC	• Process map and identify opportunities to integrate GoCC<br>• Obtain stakeholder buy-in including time to dedicate to GoCC<br>• Provide CNHs access to VA electronic health record using Community Viewer, including LST note & orders template (Project 2)
	Team members believe they are "doing a great job already"	• Use audit and feedback with comparison data to identify areas for improvement<br>• Encourage sharing of best practices among sites, with high-performing sites role-modelling for lower performing sites
	VA orders not available to CNH for Veterans discharged from VA facilities (Project 2)	• Engage inpatient VA social workers to integrate SAPO completion into the discharge process and ensure that SAPO accompanies Veterans discharged to CNH (Project 2)
Leadership	Leadership of HBPC, VA CNH team or CNH does not support conducting GoCC/completing LST template and SAPOs	• Obtain early buy-in from leadership<br>• Provide data on concordance between preferences and care<br>• Collaborate with CC and sites to incorporate the feedback reports into existing leadership meetings
Practitioners	• Desire to establish rapport before having GOCC<br>• Lack of clarity about who is responsible for conducting GoCC and completing documentation	• Refine feedback report to permit sufficient time to complete before counted as "not documented"<br>• Develop process maps for identifying responsible practitioner<br>• Engage VAMC-based discharge planners to ensure SAPO completion prior to hospital discharge (Project 2)
State laws re: SAPOs	CNH/HBPC is in a state without a well-developed POLST (SAPO) program	• Share NCEHC resources to support CNH/HBPC-directed GoCC and standardized documentation in the medical record and orders, without SAPO
Veteran or authorized surrogate	Veteran or surrogate reluctance to discuss GoCC	• Coach team members and/or CC practitioners re: engagement of Veterans/surrogates<br>• Engage VAMC palliative care team to conduct GoCC and complete LST template and SAPO

decision maker	Surrogate does not understand their role in guiding healthcare decisions for the Veteran or disagrees with decisions	• Provide team and CC with resource materials on surrogate decision making/shared decision-making to share with surrogates

NOTES: CC=clinical champion; CNH=community nursing home; GoCC=goals of care conversations; LST=Life-Sustaining Treatment; NCEHC= VA National Center for Ethics in Health Care; POLST=Physicians Order for Life-Sustaining Treatment; SAPO=State Authorize Portable Order

References Cited:

1. Ritchie MJ, Dollar KM, Miller CJ, et al. Using Implementation Facilitation to Improve Care in the Veterans Health Administration (Version 2). Veterans Health Administration, Quality Enhancement Research Initiative (QUERI) for Team-Based Behavioral Health. Veterans Health Administration, Quality Enhancement Research Initiative (QUERI) for Team-Based Behavioral Health Web site. https://www.queri.research.va.gov/tools/implementation/Facilitation-Manual.pdf. Published 2017. Accessed.

2. Carpenter J, Miller SC, Kolanowski AM, et al. Partnership to Enhance Resident Outcomes for Community Living Center Residents With Dementia: Description of the Protocol and Preliminary Findings. *J Gerontol Nurs.* 2019;45(3):21-30.

3. Banerjee SC, Manna R, Coyle N, et al. The implementation and evaluation of a communication skills training program for oncology nurses. *Transl Behav Med.* 2017;7(3):615-623.

4. Haverhals L, Manheim C, Mor V, Ersek M, Kinosian B, Lorenz K, Faricy-Anderson K, Gidwani-Marszowski R, Levy C. (2019). The experience of providing hospice care concurrent with cancer treatment in the VA. *Supportive Care in Cancer, 27,*1263-1270.

.
