# Supplementary material for: Preferences Elicited and Respected for Seriously Ill Veterans through Enhanced Decision-Making (PERSIVED): a protocol for an implementation study in the Veterans Health Administration
Source: Implement Sci Commun. 2022 Jul 20;3:78. doi: 10.1186/s43058-022-00321-2 (PMC9296899; doi:10.1186/s43058-022-00321-2)
Supplement: Supplementary file 2 — Additional file 2. PERSIVED Site Champion Description and Assessment Tool. Description: A description of responsibilities expected of clinical champions & the assessment tool to measure respondent’s confidence in areas that are important to a successful champion. [file 43058_2022_321_MOESM2_ESM.pdf]

PERSIVED Clinical Champion Description*

The goal of this QUERI-funded quality improvement (QI) project is to honor veterans' choices by engaging in goals of care conversations and documenting their preferences for life sustaining treatments in actionable, portable, durable medical orders. This project is separate from but supports the implementation of the Life-Sustaining Treatment (LST) Decisions: Eliciting, Document and Honoring Patient's Value, Goals and Preferences initiative (http://vaww.va.gov/vhapublications/).

Clinical Champions will receive regular feedback regarding their unit/facility's progress towards the completion of goals of care conversations and care planning. Clinical Champions and interested staff can also participate in virtual action planning and support calls to review data, tackle barriers together, and share best practices.

Clinical Champions should:

- be interested in and committed to supporting goals of care discussions for Veterans
- be committed to quality improvement
- be knowledgeable or willing to learn about conducting goals of care conversations, implementing the LSTDI initiative, working with VA and non-VA providers to ensure translation of veterans preferences into state authorized portable orders (e.g., POLST) that can be honored outside the VA system
- be respected by his or her peers
- possess good communication skills
- possess in-depth knowledge about the institution and organizational culture

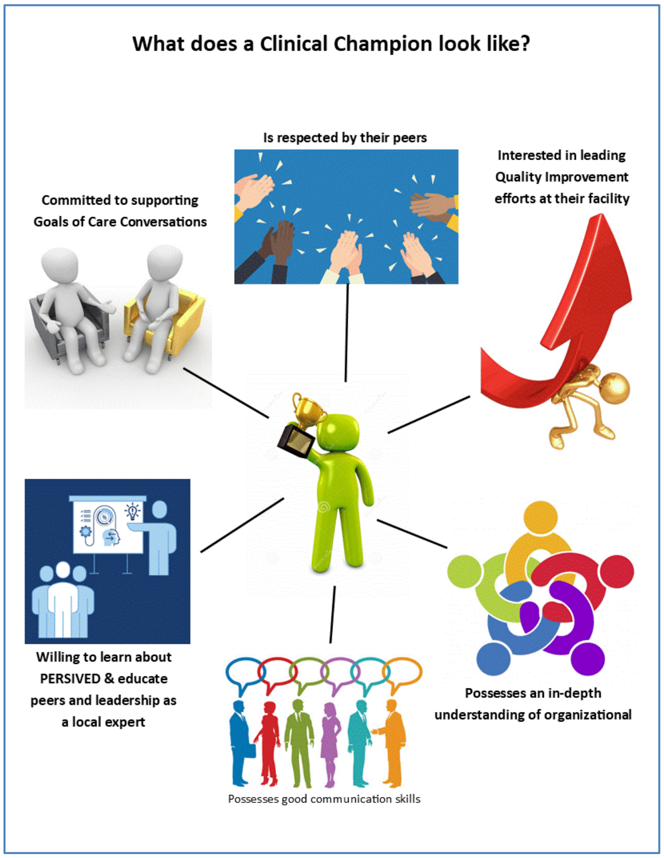

The specific roles of Clinical Champions are to:

- Educate
  - attend the LST initiative training session, if appropriate
  - serve as a local expert and resource to staff about PERSIVED
  - relay information among local VA leadership/supervisors, HBPC and CNH teams and non-VA providers and staff, such as those in community nursing homes and hospice/palliative care organizations

- Advocate
  - keep facility staff and leadership engaged in and committed to the QI project
  - communicate the importance of the QI project in improving the quality of life for veterans and their families
  - foster participation in the QI project among VA and non-VA providers and staff

- Build relationships
  - cultivate relationships with VA and on VA staff and providers that promote and support the partnership

    - communicate with VA and none VA staff and leadership about their role
    - be actively engaged in activities (e.g. face-to-face meetings, webinars, and phone conferences) related to the project

- Navigate boundaries
    - keep in close contact with VA and VA provider staff and leadership, especially those who might question the value of the QI project
    - openly communicate with team members about challenges they encounter
    - brainstorm about opportunities to overcome real or perceived barriers

Why should you be a Clinical Champion?

The main reason to become a Clinical Champion is to support and promote patient and family-centered care. Becoming a Clinical Champion can assist staff in their professional goals to assume greater responsibility in the improvement of patient centered care. It also allows staff members to evolve professionally as the VA transforms the way LST preferences and decisions are documented nation-wide.

*Adapted from Carpenter et al. Partnership to Enhance Resident Outcomes for Community Living Center Residents with Dementia: Description of the protocol and preliminary findings**.** Journal of Gerontological Nursing, 2019.

Clinical Champion Assessment of Effectiveness Tool

Clinical Champion Assessment of Effectiveness Tool				
**For the items below, please indicate the level of confidence you have for your effectiveness:**				
Item		Not at all confident	Somewhat confident	Very confident
1	I can explain to stakeholders (including facility leadership, clinical teams, and community partners) the purpose, implementation methods, and outcomes of the EBPs of the PERSIVED program.			
2	I am knowledgeable about the life-sustaining treatment decisions initiative (LSTDI) and or state authorized portable orders (SAPOs).			
3	I can effectively role model and guide clinicians in conducting goals of care conversations.			
4	I can accurately role model and guide clinicians in completing an LST template and/or SAPO.			
5	I am knowledgeable about the life-sustaining treatment decisions initiative (LSTDI) and or state authorized portable orders (SAPOs).			
6	I am able to communicate accurate and complete information contained in the feedback reports to clinicians and leaders.			
7	I can help clinical teams develop SMART goals to improve their performance in completing LST templates/SAPOs			
8	I know where to find resources related to the LSTDI and/or to SAPOs.			
9	I can communicate to leaders, practitioners, and other team members why it is important to conduct a goals of care conversation and document Veterans' preferences in durable orders.			
10	I can communicate to practitioners and other team members how to locate LST templates and/or SAPOs in the medical record.			
11	I have the support that I need from facility leaders to fulfill my responsibilities as clinical champion for this project.			
12	I have the support that I need from practitioners to promote the conduct of goals of care conversations and completion of LST template/SAPOs.			
13	I can communicate to Veterans and/or their family surrogates the importance of engaging in goals of care conversations and documenting the Veteran's preference is for care.			
14	I have the knowledge and skills needed to refine process maps related to this evidence-based practice.			
15	I can help teams identify barriers and facilitators to implementing this evidence-based practice.			

.
