# Supplementary material for: Preferences Elicited and Respected for Seriously Ill Veterans through Enhanced Decision-Making (PERSIVED): a protocol for an implementation study in the Veterans Health Administration
Source: Implement Sci Commun. 2022 Jul 20;3:78. doi: 10.1186/s43058-022-00321-2 (PMC9296899; doi:10.1186/s43058-022-00321-2)

[redacted] CNH Process Map: Veterans receiving VA-paid LTC at a Community Nursing Home

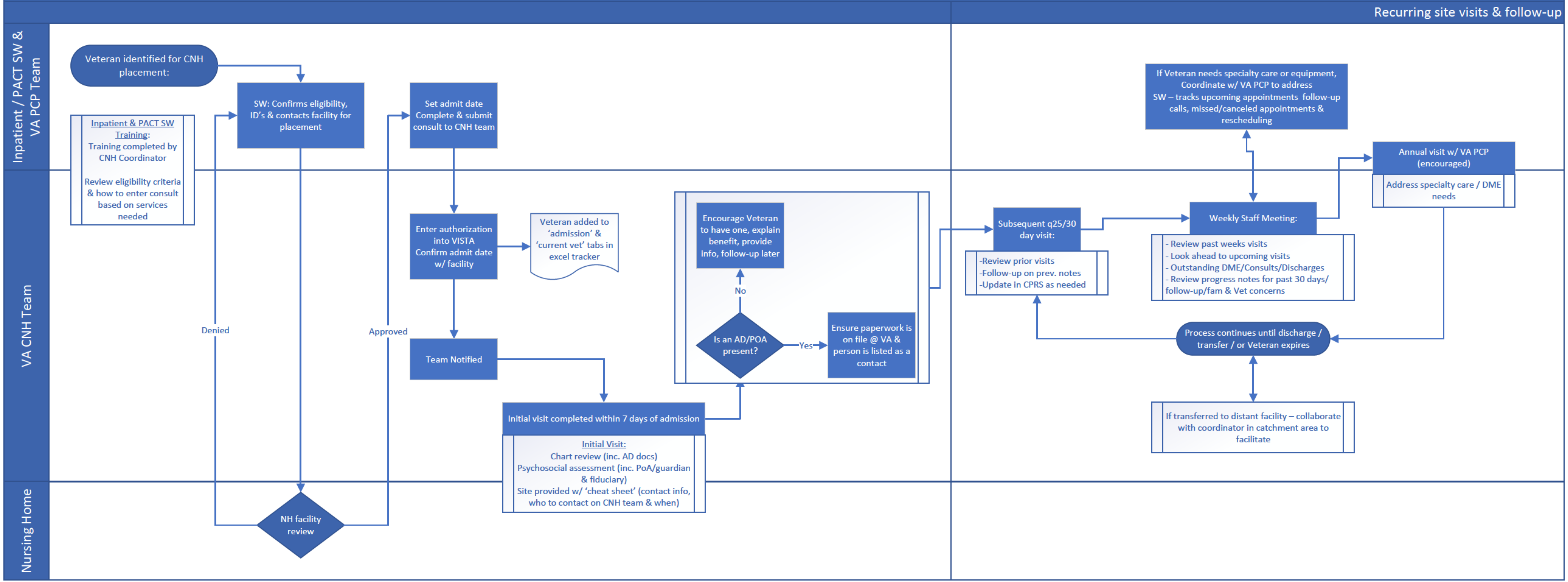

Supplement: Supplementary file 3 — Additional file 3. Example of Process Map. Description: A process map (redacted) developed with a participating VA CNH program during the pre-implementation phase. [file 43058_2022_321_MOESM3_ESM.pdf]
