# Supplementary material for: Preferences Elicited and Respected for Seriously Ill Veterans through Enhanced Decision-Making (PERSIVED): a protocol for an implementation study in the Veterans Health Administration
Source: Implement Sci Commun. 2022 Jul 20;3:78. doi: 10.1186/s43058-022-00321-2 (PMC9296899; doi:10.1186/s43058-022-00321-2)
Supplement: Supplementary file 4 — Additional file 4. Example of Feedback Report. Description: An example of the feedback reports that are generated each month for participating sites. [file 43058_2022_321_MOESM4_ESM.pdf]

Documented Life-Sustaining Treatment Decisions

Feedback Report

May 18, 2022

Mock Site

The figure and table below display the number and percentages of completed LST templates for Veterans at Mock Site .
Figure 1 shows the percentage of patients with prior documentation along with those completed in the last month. These counts are also shown in Table 1 along with the total number of patients at the site and the number and percent without documentation by month. The rate that you targeted in your SMART (Specific, Measurable, Attainable, Relevant, Time-based) goal is indicated by the blue reference line.

Figure 1: Documentation rates by month

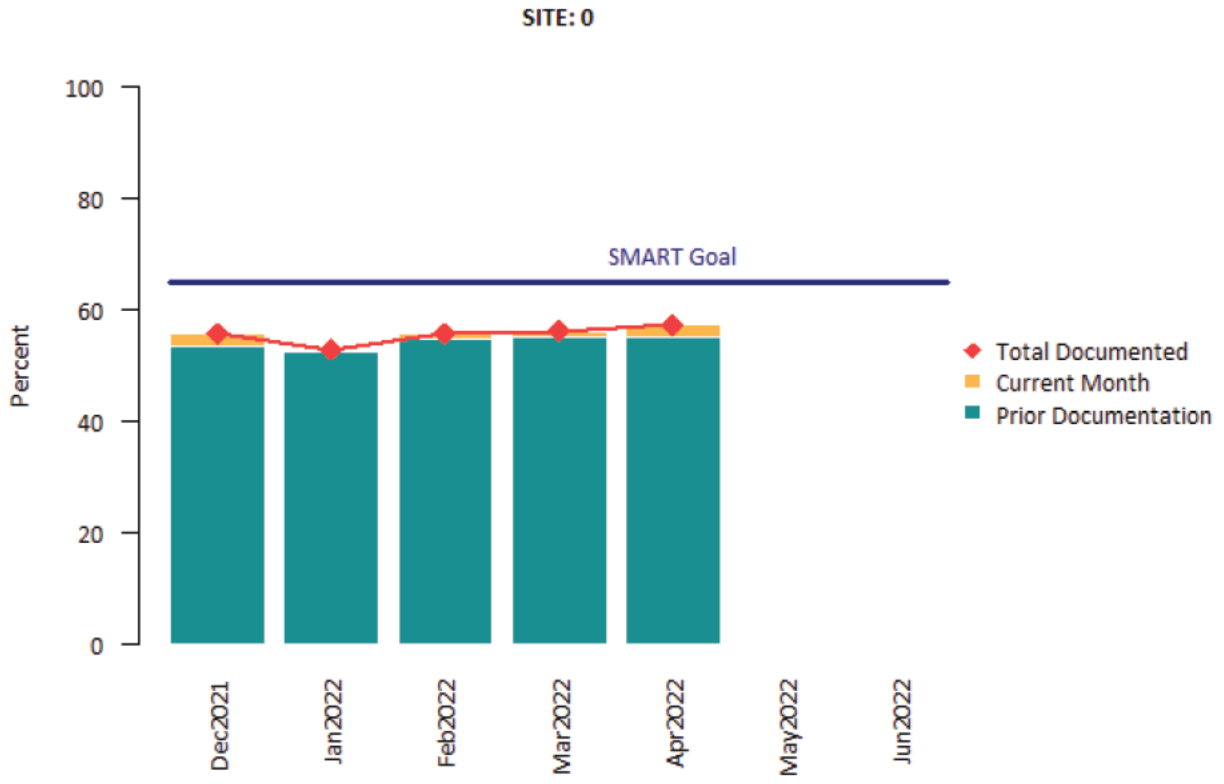

Table 1: Documentation rates by month

Month/Year	Not Documented	Current Month	Prior Documentation	Total
Dec2021	84 (44.68%)	4 (2.13%)	102 (54.26%)	190
Jan2022	92 (46.94%)	1 (0.51%)	103 (52.55%)	196
Feb2022	88 (44.22%)	2 (1.01%)	109 (54.77%)	199
Mar2022	90 (43.69%)	2 (0.97%)	114 (55.34%)	206
Apr2022	91 (42.52%)	5 (2.34%)	118 (55.14%)	214

Table 2: Documentation rates by month (Newly Admitted)

Month/Year	Not Documented	Current Month	Prior Documentation	Total
Feb2022	9 (45%)	1 (5%)	10 (50%)	20
Mar2022	9 (40.91%)	1 (4.55%)	12 (54.55%)	22
Apr2022	9 (36%)	2 (8%)	14 (56%)	25

Please review this information with your entire team to think about strategies to increase the number of goals of care conversations (GoCCs) and completed LST order templates for veterans in your program.

As you review, please remember these important points:

- These data come from a roster provided by the HBPC team. The data and roster reflect the census at that point in time.
- Only LST templates with completed answers for all required LST template items were counted.
- If a Veteran had more than one LST template completed in a month, only the last template was counted.
- Each Veteran was counted only once even if they had multiple stays.
- You can find more information about the LSTDI and its implementation at: http://www.ethics.va.gov/LST.asp

Thank you for your efforts to ensure that veterans' goals of care and treatment preferences are discussed and documented.

If you have any questions about this report or the accuracy of the data, please contact your Site Champion or PERSIVED facilitation lead.

Site Champions
Champion1 (champion.1@va.gov)
Champion2 (champion.2@va.gov)

PERSIVED Facilitation Leads
[redacted]
[redacted]
